# Causes and consequences of pattern diversification in a spatially self-organizing microbial community

**DOI:** 10.1038/s41396-021-00942-w

**Published:** 2021-03-04

**Authors:** Felix Goldschmidt, Lea Caduff, David R. Johnson

**Affiliations:** 1 Department of Environmental Systems Science, Swiss Federal Institute of Technology (ETH), Zürich, Switzerland; 2 Department of Environmental Microbiology, Swiss Federal Institute of Aquatic Science and Technology (Eawag), Dübendorf, Switzerland

**Keywords:** Microbial ecology, Microbial ecology, Biofilms

## Abstract

Surface-attached microbial communities constitute a vast amount of life on our planet. They contribute to all major biogeochemical cycles, provide essential services to our society and environment, and have important effects on human health and disease. They typically consist of different interacting genotypes that arrange themselves non-randomly across space (referred to hereafter as spatial self-organization). While spatial self-organization is important for the functioning, ecology, and evolution of these communities, the underlying determinants of spatial self-organization remain unclear. Here, we performed a combination of experiments, statistical modeling, and mathematical simulations with a synthetic cross-feeding microbial community consisting of two isogenic strains. We found that two different patterns of spatial self-organization emerged at the same length and time scales, thus demonstrating pattern diversification. This pattern diversification was not caused by initial environmental heterogeneity or by genetic heterogeneity within populations. Instead, it was caused by nongenetic heterogeneity within populations, and we provide evidence that the source of this nongenetic heterogeneity is local differences in the initial spatial positionings of individuals. We further demonstrate that the different patterns exhibit different community-level properties; namely, they have different expansion speeds. Together, our results demonstrate that pattern diversification can emerge in the absence of initial environmental heterogeneity or genetic heterogeneity within populations and can affect community-level properties, thus providing novel insights into the causes and consequences of microbial spatial self-organization.

## Introduction

Surface-attached microbial communities such as multispecies biofilms and cell aggregates are omnipresent on our planet. They contribute to every major biogeochemical cycle, provide essential services to our society and environment, and have important effects on human health and disease [1–4]. They are typically composed of different genotypes that interact with each other and arrange themselves non-randomly across space (referred to hereafter as spatial self-organization [5–7]). Spatial self-organization can result in the emergence of fascinating and intriguing patterns [8–20] and can also be an important determinant of community-level properties [7]. For example, spatial self-organization can affect community-level productivity [11, 21–23], enable otherwise impermissible metabolic processes to occur [24–26], bestow resistance or resilience to environmental perturbations [27–29] and invaders [30, 31], and modulate evolutionary processes [18, 32–35]. Elucidating the underlying determinants of spatial self-organization is therefore important for our basic understanding of the functioning, ecology, and evolution of microbial communities and for modulating and controlling community-level properties [36].

**Fig. 1:**
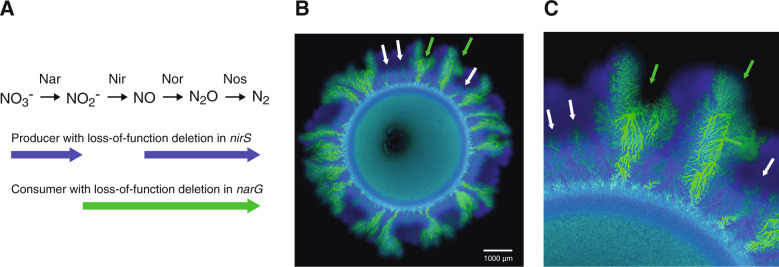
Synthetic two-strain cross-feeding microbial community used in this study. **A** The experimental system consists of two isogenic mutants of the bacterium *Pseudomonas stutzeri*. The producer contains a single loss-of-function deletion in the *nirS* gene and can reduce nitrate (NO_3_^−^) but not nitrite (NO_2_^−^). The consumer contains a single loss-of-function deletion in the *narG* gene and can reduce nitrite but not nitrate. The strains cross-feed nitrite when grown together with an exogenous supply of nitrate as the growth-limiting substrate. Definitions: Nar nitrate reductase, Nir nitrite reductase, Nor nitric oxide reductase, Nos nitrous oxide reductase. Thick colored arrows indicate the metabolic processes performed by each strain. **B** Four weeks of expansion of the two-strain cross-feeding microbial community when supplied with nitrate as the growth-limiting substrate. The producer expressed the cyan fluorescent protein-encoding *ecfp* gene (blue) while the consumer expressed the green fluorescent protein-encoding *egfp* gene (green). The inner circular region containing well-mixed producer and consumer cells is the inoculation area while the outer ring-like region containing spatially organized patterns of producer and consumer cells is the expansion area. The initial producer and consumer proportions were 0.5. White arrows indicate representative “producer first” patterns and green arrows indicate representative “consumer first” patterns. **C** Magnification of the expansion area.

Given a set of genotypes and an initial environment that is spatially homogeneous, we might expect a single pattern of spatial self-organization to emerge at particular length and time scales, where the pattern is determined by the initial environmental conditions and the interactions that occur between different genotypes. This expectation, however, is not always realized. We analyzed patterns of spatial self-organization that emerged as a synthetic two-strain cross-feeding community expanded across an initial environment that was spatially homogeneous [18, 20, 37] (Fig. 1). The two strains are genetically engineered isogenic mutants that interact via cross-feeding when grown together in an environment containing nitrate (NO_3_^−^) as the growth-limiting resource, where one strain reduces nitrate to nitrite (NO_2_^−^) (referred to hereafter as the producer) while the other strain reduces the released nitrite (referred to hereafter as the consumer) [18, 38] (Fig. 1A and Supplementary Table S1). Using this system, we found that two different patterns of spatial self-organization emerged simultaneously (white and green arrows; Fig. 1B, C) [18]. One pattern is “producer first”, where the producer lies at the expansion frontier and expands before the consumer (Fig. 1A, B, white arrows) [18]. The consumer then forms fractal-like branching patterns as it advances through the biomass formed by the producer (Fig. 1A, B, white arrows) as explored previously [18, 20]. A defining feature of this pattern is that the orientation of the branching is primarily towards the expansion frontier (white arrows; Fig. 1C). The second pattern is “consumer first”, where the consumer lies at the expansion frontier and the producer and consumer expand at approximately the same time (green arrows; Fig. 1C) [18]. A defining feature of this pattern is that the orientation of the branching is in the opposite direction and primarily toward the origin of expansion (green arrows; Fig. 1C). Importantly, the two different patterns emerged at the same length and time scales and from the very origin of expansion (Fig. 1C) [18]. We provide multiple lines of evidence that the “producer first” and “consumer first” patterns emerge as a consequence of the genetically engineered cross-feeding interaction in the Supplementary Text and Supplementary Figs. S1, S2.

Why did two different patterns of spatial self-organization emerge at the same length and time scales when the initial environment was spatially homogeneous? In other words, what caused the observed pattern diversification? One hypothesis is that there was genetic heterogeneity within populations. Genetic variants might have been present that had different biological traits, and thus were subject to different deterministic rules, and these variants caused the observed pattern diversification. A second hypothesis is that there was nongenetic heterogeneity within populations. Nongenetic variants might have been present that again had different biological traits, and thus were subject to different deterministic rules, and these variants caused the observed pattern diversification. Nongenetic heterogeneity could be caused by at least three different processes. The first is neighborhood effects, which can occur when individuals are not distributed uniformly across a surface. For example, if individuals are distributed randomly across a surface, there will be local differences in the distances between neighboring individuals. This, in turn, could affect local environmental conditions and the mechanical and biological interactions occurring between cells, and consequently modulate local phenotypic properties [39]. The second is stochastic phenotypic heterogeneity within populations, which occurs when different individuals within a genetically homogeneous population express different phenotypic traits due to mechanisms such as stochastic gene expression [40–44]. Stochastic phenotypic variants might therefore be present that express different biological traits. The third is demographic effects, such as differences in cell age or history [45, 46]. Distinguishing between neighborhood effects, stochastic phenotypic heterogeneity, and demographic effects can be exceedingly difficult, as all three can create phenotypic variants within populations.

The objective of this study was to investigate whether genetic or nongenetic heterogeneity within populations caused the observed diversification in patterns of spatial self-organization (Fig. 1B, C). To accomplish this, we used the same synthetic two-strain cross-feeding microbial community that we have been investigating [18, 20, 38, 47–49] (Fig. 1). We first tested whether genetic heterogeneity caused the observed pattern diversification. We next tested whether nongenetic heterogeneity caused the observed pattern diversification. More specifically, we tested whether neighborhood effects alone could explain our experimental observations. Finally, we tested whether the different patterns have different community-level properties.

## Materials and methods

### Bacterial strains

We previously described in detail all of the strains and growth conditions used in this study [18, 38, 47] (see Supplementary Text). We further summarized the biological traits of the strains in Fig. 1A and all of their genetic modifications in Supplementary Table S1. Briefly, we previously constructed two isogenic strains of the bacterium *Pseudomonas stutzeri* A1501. The producer contains a single loss-of-function deletion in the nitrite (NO_2_^−^) reductase-encoding *nirS* gene and can reduce nitrate (NO_3_^−^) but not nitrite [38] (Fig. 1A, Supplementary Table S1). The consumer contains a single loss-of-function deletion in the nitrate reductase-encoding *narG* gene and can reduce nitrite but not nitrate [38] (Fig. 1A and Supplementary Table S1). When the two strains are grown together with an exogenous supply of nitrate as the growth-limiting substrate, they cross-feed nitrite, where the producer produces nitrite and the consumer reduces nitrite [38, 48]. In addition, all of the strains contain a single loss-of-function deletion in the *comA* gene [38] (Supplementary Table S1), which prevents natural transformation [50] and minimizes the probability that the two strains will recombine with each other when grown together. Finally, each strain contains a different isopropyl β-D-1-thiogalactopyranoside (IPGT)-inducible fluorescent protein-encoding gene (*ecfp* encoding for cyan fluorescent protein, *egfp* encoding for green fluorescent protein, or *echerry* encoding for red fluorescent protein [51]) [18, 48] (Supplementary Table S1), which enables us to distinguish and quantify the different strains when grown together. We reported all of the methods used to construct the strains in detail elsewhere [18, 38, 47].

### Range expansion experiments

We performed range expansion experiments using a protocol that we described previously [18, 20, 49], which is a modified anaerobic version of a protocol described elsewhere [9]. Briefly, we first grew the producer and consumer separately in aerobic liquid lysogeny broth (LB) medium overnight. We next diluted the liquid cultures such that the producer and consumer had equivalent optical densities at 600 nm (OD_600_). Both strains are derived from the same ancestor and have the same optical properties at this wavelength; equivalent optical densities therefore approximate to equivalent cell numbers. We then mixed the producer and consumer together at defined initial proportions, where each strain contains a different fluorescent protein-encoding gene (i.e., the producer containing the *ecfp* gene mixed with the consumer containing the *egfp* gene or vice versa). Finally, we transferred the mixtures into a glove box (Coy Laboratory Products, Grass Lake, MI) filled with an anaerobic nitrogen (N_2_):hydrogen (H_2_) (97%:3%) atmosphere, deposited a single 2 µl aliquot from each mixture onto the middle of a separate anaerobic LB agar plate amended with 100 µM IPTG and 1 mM nitrate (NO_3_^−^) as the growth-limiting substrate, and incubated the anaerobic LB agar plates for 4 weeks at room temperature. We reported a complete description of the methods to prepare anaerobic LB agar plates elsewhere [18]. In this study, we set the pH of the LB agar plates to 7.5. At this pH, the cross-fed intermediate nitrite (NO_2_^−^) has no quantifiable growth-inhibiting effects [38], and the interaction between the producer and consumer is therefore approximately commensal (i.e., the consumer depends on the producer to provide its growth supporting substrate nitrite while the producer does not depend on the consumer). After 4 weeks of incubation, we removed the LB agar plates from the glove box and exposed them to ambient air for 1 h to induce maturation of the fluorescent proteins [52]. We then imaged the resulting colonies using a Leica TCS SP5 II confocal microscope (Leica Microsystems, Wetzlar, Germany), analyzed the images in ImageJ (https://imagej.net), and delineated and quantified the number of “producer first” and “consumer first” patterns as described in detail elsewhere [18, 20]. We measured colony radii using Fiji plugins (https://fiji.sc). Briefly, we selected the expansion edge using the “wand” tool, fit an ellipse to the expansion edge using the “fit ellipse” tool, and used the *x*-axis (ellipse length) and *y*-axis (ellipse width) measurements of the fitted ellipse to calculate the radius, where the radius = (*x* + *y*)/4.

### Test for heritability

We tested whether the “consumer first” pattern of spatial self-organization is heritable. To achieve this, we obtained a set of isolates purified from prior ‘consumer first’ patterns. We first used a Wild M5A stereo microscope (Heerbrugg, Switzerland) to identify a set of spatially delineated ‘consumer first’ patterns and used a separate sterile toothpick to remove cells from each of these patterns. We next streaked the sterile toothpicks onto separate LB agar plates supplemented with 100 µM IPTG and incubated the plates for 24 h at 30 °C. We then placed the LB agar plates under a confocal microscope (Leica Microsystems, Wetzlar, Germany) and used the expression of the different fluorescent proteins to identify individual colonies that consisted of only the producer or consumer (i.e., we avoided individual colonies that expressed both fluorescent proteins, which indicates that those colonies were not derived from single cells). We next streaked the identified colonies a second time onto new LB agar plates to obtain a set of isolates purified from prior “consumer first” patterns. Finally, we randomly selected a pair of isolates of the producer and consumer from the same “consumer first” pattern, mixed them together at an initial producer proportion of 0.5 (i.e., an initial consumer proportion of 0.5) and performed ten new range expansion experiments as described above. If the “consumer first” pattern were heritable, we expected the number of “consumer first” patterns to increase during the second range expansion experiment when using the pair of isolates purified from the prior ‘consumer first’ pattern.

### Individual-based model simulations

We previously described the individual-based reaction-diffusion model in detail elsewhere [20], and we provide further details on its setup for this study in the Supplementary Text. The original model and its adaptions to microbial range expansion are described in detail elsewhere [17, 53]. Briefly, individual cells are modeled as spheres on a nutrient grid. The cells grow in size and divide after reaching a threshold size. After division, the cells rearrange themselves in space using a shoving algorithm. Growth of the producer and consumer are modeled using Monod-type formulations (Supplementary Text). The consumption of nitrate (NO_3_^−^) and nitrite (NO_2_^−^) are related to growth of the producer and consumer, respectively, using yield coefficients (Supplementary Text). The production of nitrite is equivalent to the consumption of nitrate by the producer as per stoichiometry. To simulate our experimental findings, we modified the initial producer proportion while maintaining a fixed total initial number of cells. Note that due to computational constraints, the total number of simulated cells is much lower than in the experiments. We report the parameter values in Supplementary Table S2.

### Statistics and data analysis

We used the two-sided *t* test to test whether the numbers of “consumer first” patterns that emerged per range expansion differ from null hypotheses. We used the *F*-test to test whether the expansion speeds differ between treatments. We fit linear and Poisson regression models to the data and calculated summary statistics in the R environment using core functions [54].

## Results

### The “consumer first” pattern of spatial self-organization is the minority pattern

We first determined which of the two patterns of spatial self-organization (i.e., the “producer first” or “consumer first” pattern (Fig. 1B, C)) is the minority pattern. We reasoned that the minority pattern is the one likely to be caused by genetic or nongenetic variants. When we performed range expansion experiments using equivalent initial cell densities of the producer and consumer (i.e., initial producer and consumer proportions of 0.5), the “consumer first” pattern was clearly the minority pattern (Fig. 1B). Among nine independent replicates, we observed mean numbers of 64 “producer first” patterns (SD = 9, *n* = 9) and 20 “consumer first” patterns per range expansion (SD = 4, *n* = 9), and the mean number of “producer first” patterns was significantly greater than the mean number of “consumer first“ patters (two-sample two-sided *t* test; *P* = 1 × 10^−6^, *n* = 9). Overall, “consumer first” patterns accounted for 24% (SD = 4%, *n* = 9) of the total number of patterns per range expansion. We therefore conclude that the “consumer first” pattern is indeed the minority pattern, and thus the pattern likely to be caused by genetic or phenotypic variants.

### The number of “consumer first” patterns depends on initial cell densities

If the “consumer first” pattern were caused by genetic variants, then the number of “consumer first” patterns that emerge per range expansion should depend on the initial cell densities of the producer or consumer. For example, if a genetic variant of the consumer causes the emergence of the “consumer first” pattern, then increasing the initial cell density of the consumer should increase the number of the causative variants of the consumer, and thus promote the emergence of more “consumer first” patterns.

**Fig. 2:**
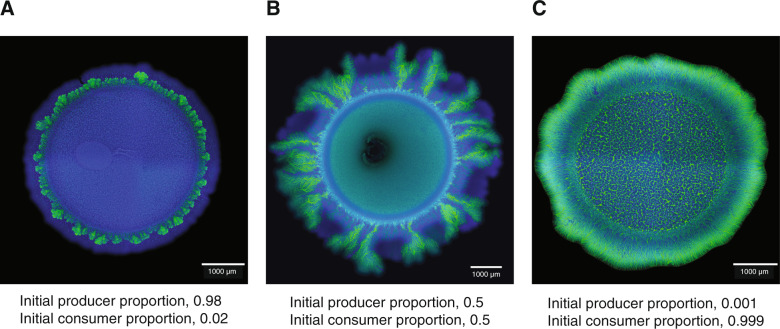
Effect of the initial producer proportion on the number of “consumer first” patterns that emerge per range expansion after 4 weeks. The producer expressed the cyan fluorescent protein-encoding *ecfp* gene (blue) while the consumer expressed the green fluorescent protein-encoding *egfp* gene (green). Initial producer proportions include (**A**) 0.98, (**B**) 0.5, and (**C**) 0.001. The total initial cell densities of producer and consumer were identical across all of the tested initial producer proportions.

**Fig. 3:**
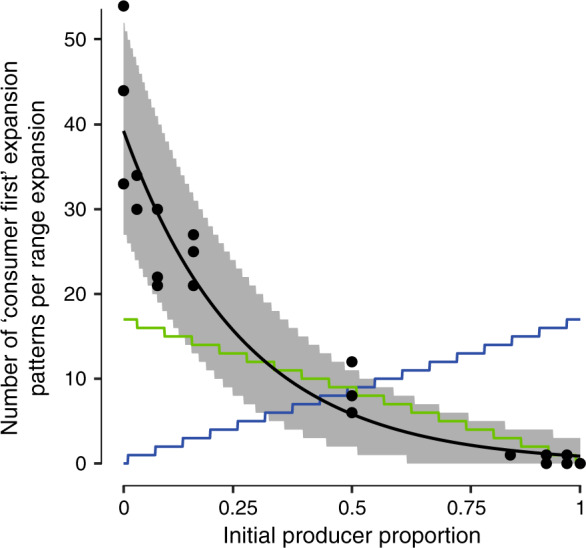
Effect of the initial producer proportion on the number of “consumer first” patterns that emerge per range expansion after 4 weeks. Each data point is the number of “consumer first” patterns that emerged for an independent range expansion. The black line is the fit of a Poisson regression model to the data. The gray area is the 95% confidence interval of Poisson distributions with *λ* = predicted value of the Poisson regression fit. The green line is the expected relationship between the number of “consumer first” patterns and the initial producer proportion if the “consumer first” pattern were caused by genetic variants of the consumer. The blue line is the expected relationship between the number of “consumer first” patterns and the initial producer proportion if the “consumer first” pattern were caused by genetic variants of the producer. The total initial cell densities of producer and consumer were identical across all of the tested initial proportions.

To test this, we varied the initial producer proportion while holding the total initial cell density of the producer and consumer constant, thus allowing us to avoid potential confounding effects that may result from modifying the total initial cell density. We then quantified the mean number of “consumer first” patterns that emerged per range expansion as a function of the initial producer proportion. When we tested an initial producer proportion of 0.5 (i.e., an initial consumer proportion of 0.5), we observed the characteristic emergence of the two different patterns, where the “consumer first” pattern was the minority pattern (Fig. 2B). When we tested an initial producer proportion of 0.98 (i.e., an initial consumer proportion of 0.02), the “consumer first” pattern completely disappeared while the “producer first” pattern occupied the entire expansion area (Fig. 2A). In contrast, when we tested an initial producer proportion of 0.001 (i.e., an initial consumer proportion of 0.999), the “producer first” pattern completely disappeared while the “consumer first” pattern occupied the entire expansion area (Fig. 2C). We then repeated the experiment across a range of initial producer proportions and observed a decreasing monotonic relationship between the mean number of “consumer first” patterns that emerged per range expansion and the initial producer proportion (Fig. 3). We could model the decreasing relationship with a Poisson regression using the natural logarithm as the link function (intercept = 3.76, slope = −4.15, *P* for both parameters = 2 × 10^−16^, *n* = 9) (black line; Fig. 3). Thus, the number of “consumer first” patterns that emerge per range expansion does indeed depend on the initial cell densities of the producer and consumer.

### Genetic variants as a cause of the observed pattern diversification

While we found that the number of “consumer first” patterns that emerge per range expansion depends on the initial cell densities of the producer and consumer (Fig. 3), the log-linear form of the decreasing relationship is inconsistent with the “consumer first” pattern being caused by genetic variants. Consider initial producer proportions of 0.02. 0.05, or 0.1 (i.e., initial consumer proportions of 0.98, 0.95, or 0.9). At these initial producer proportions, the initial cell density of the consumer is approximately twofold greater (1.96-, 1.90-, and 1.8-fold, respectively) than that for an initial producer proportion of 0.5 (i.e., an initial consumer proportion of 0.5). If genetic variants of the consumer cause the emergence of the “consumer first” pattern, we would therefore expect approximately twofold more “consumer first” patterns to emerge per range expansion. More generally, we would expect the number of “consumer first” patterns that emerge per range expansion to decrease linearly as the initial producer proportion increases (i.e., as the initial consumer proportion decreases) (Fig. 3, green line) (see the Supplementary Text for the formulation of this expectation). We did not observe either of these expectations. First, at initial producer proportions of 0.02, 0.05, or 0.1 (i.e., initial consumer proportions of 0.98, 0.95, or 0.9), the number of “consumer first” patterns was not approximately twofold greater than at an initial producer proportion of 0.5 (i.e., an initial consumer proportion of 0.5). Instead, it was three to fivefold greater (Fig. 3) (one-sample two-sided *t* test; *P* = 6 × 10^−5^, *n* = 3). Second, we experimentally observed a decreasing log-linear relationship (black line; Fig. 3) rather than the expected decreasing linear relationship (green line; Fig. 3) between the number of “consumer first” patterns that emerged per range expansion and the initial producer proportion. Thus, we conclude that genetic variants of the consumer are unlikely to cause the emergence of the two patterns of spatial self-organization.

Our analysis above assumes that the “consumer first” pattern is caused by genetic variants of the consumer. However, it is plausible that the “consumer first” pattern is instead caused by genetic variants of the producer. The form of the relationship between the number of “consumer first” patterns that emerged per range expansion and the initial cell densities of the producer and consumer, however, is again inconsistent with this hypothesis (Fig. 3). If genetic variants of the producer cause the emergence of the “consumer first” pattern, then we would expect the number of “consumer first” patterns that emerge per range expansion to increase as the initial producer proportion increases (blue line; Fig. 3). Stated alternatively, increasing the initial producer proportion will increase the abundance of the causative variants of the producer, and thus increase the number of “consumer first” patterns that emerge. However, we observed the opposite outcome, where the number of “consumer first” patterns that emerged per range expansion decreased as the initial producer proportion increased (black line; Fig. 3). Thus, we conclude that genetic variants of the producer are also unlikely to cause the emergence of the two different patterns of spatial self-organization.

While the above analyses provide circumstantial evidence that genetic variants do not cause the simultaneous emergence of the two different patterns of spatial self-organization, we sought to provide more conclusive evidence of this by testing whether the “consumer first” pattern is heritable. To achieve this, we obtained a collection of isolates purified from prior “consumer first” patterns. We then mixed the isolates together (one producer with one consumer; initial producer and consumer proportions of 0.5) and repeated the range expansion experiment. Finally, we counted the numbers of “consumer first” patterns that emerged during the second range expansion and compared the numbers to those for pairs of the ancestral strains (producer and consumer). If the emergence of the “consumer first” pattern were heritable, we would expect more “consumer first” patterns when using pairs of isolates purified from prior “consumer first” patterns.

We found that pairs of isolates (producer and consumer) purified from prior “consumer first” patterns do not behave differently when compared to pairs of the ancestral strains (producer and consumer). Among ten independent range expansions for a pair of isolates (producer and consumer) purified from a prior “consumer first” pattern, we found that the “consumer first” pattern completely covered the expansion area for one of the ten replicates (Supplementary Fig. S3a). However, among ten independent range expansions for the pair of ancestral strains (producer and consumer), we found that the “consumer first” pattern also completely covered the expansion area for one of the ten replicates (Supplementary Fig. S3b). Overall, among the remaining nine independent range expansions, we did not detect more “consumer first” patterns per range expansion for the pair of isolates (producer and consumer) purified from a prior “consumer first” pattern than for the pair of ancestral strains (producer and consumer) (two-sample two-sided *t* test; *P* = 0.27, *n* = 9). Moreover, we sequenced the genomes of four producer isolates and four consumer isolates purified from prior “consumer first” patterns and found only one putative genetic difference in a single consumer isolate when compared to their respective ancestors (Supplementary Text and Supplementary Table S3). Thus, the “consumer first” pattern is not heritable, and its emergence is therefore not caused by genetic variants.

### Neighborhood effects as a cause for the observed pattern diversification

**Fig. 4:**
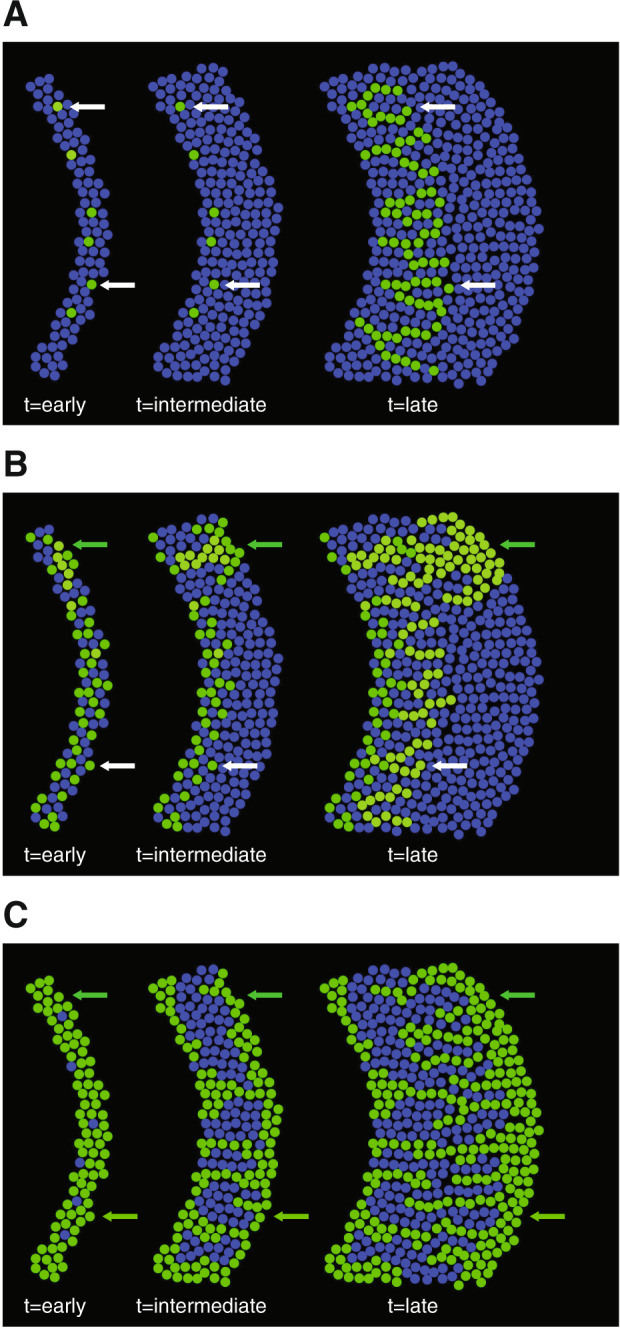
Conceptual model for how local differences in the initial spatial positionings of individual cells could promote diversification in patterns of spatial self-organization. The producer is blue while the consumer is green. The initial producer proportion is (**A**) approximating to 1, (**B**) 0.5, or (**C**) approximating to 0. White arrows indicate “producer first” patterns and green arrows indicate “consumer first” patterns. The horizontal panels from left to right depict pattern formation over time.

If the simultaneous emergence of the two different patterns of spatial self-organization is not caused by genetic variants, what then could be the cause? We argue that one plausible cause is neighborhood effects that emerge due to local differences in the initial spatial positionings of otherwise identical individuals. Consider random initial distributions of producer and consumer cells across a surface (initial producer and consumer proportions of 0.5; Fig. 4B). At some spatial locations, producer cells may initially lie sufficiently close to the expansion frontier such that the consumer cells do not physically impede their expansion (white arrow; Fig. 4B). The producer cells would then expand first while the consumer cells would expand afterwards, giving rise to the “producer first” pattern (white arrow; Fig. 4B). However, at other spatial locations, producer cells may initially lie behind a cluster of consumer cells such that the consumer cells physically impede the expansion of the producer cells (green arrow; Fig. 4B). Indeed, we observed this experimentally at an intermediate timepoint of expansion (Supplementary Fig. S4). These clusters of consumer cells can occur purely as a consequence of the random initial spatial positionings of those cells, a process known as Poisson clumping [55]. The producer cells would then shove the consumer cells forward as they expand, giving rise to the “consumer first” pattern (green arrow; Fig. 4B). This hypothesis assumes that cell shoving is the dominant form of cell movement in the densely packed expanding microbial colonies produced by our synthetic microbial community, which is an assumption supported by numerous experimental and theoretical investigations [17, 53, 56–60].

Importantly, this hypothesis is qualitatively consistent with our experimentally observed relationship between the number of “consumer first” patterns that emerge per range expansion and the initial proportions of the producer and consumer (Fig. 3). If producer cells initially far outnumber consumer cells, then the “consumer first” pattern should become less numerous (Fig. 4A). This is because there are fewer consumer cells present to create the necessary cell clusters that physically impede the expansion of the producer cells, and the producer cells can therefore expand immediately giving rise to the “producer first” pattern (Fig. 4A). In contrast, if the consumer cells initially far outnumber producer cells, then the “consumer first” pattern should become more numerous (Fig. 4C). This is because there are more consumer cells present to create the necessary cell clusters that physically impede the expansion of the producer cells, and the producer cells must therefore shove the consumer cells forward giving rise to the “consumer first” pattern (Fig. 4C).

This hypothesis is also consistent with quantitative features of our experimentally observed relationship between the number of “consumer first” patterns that emerge per range expansion and the initial proportions of the producer and consumer (Fig. 3). The initial spatial distributions of producer and consumer cells can be thought of as realizations of a Poisson point process. Such a process by chance produces clusters of consumer cells whose occurrence is described by a Poisson distribution with mean and variance λ. The mean number of consumer clusters and variance depend on the initial proportion of the consumer in a log-linear manner. As the initial proportion of the consumer increases, the probability for a consumer cluster to occur also increases. This, in turn, increases the probability that a “consumer first” pattern will form and increases the variance in the expected number of “consumer first” patterns. We found that this Poisson process accurately captures key features of our experimental data. First, the relationship between the number of “consumer first” patterns and the initial consumer proportion is modeled very well by a Poisson regression (Fig. 3). Second, the variance in the number of “consumer first” patterns increases as the experimentally observed number of “consumer first” patterns increases (Fig. 3). Third, the 95% confidence intervals of the Poisson distributions with *λ* equal to the predicted value of the Poisson regression matches the spread of the experimentally observed number of “consumer first” patterns (Fig. 3). In summary, the log-linear shape and the increasing variance of the data are thus consistent with our hypothesis that Poisson clumping due to the random initial spatial positionings of individuals causes the ‘consumer first’ pattern and promotes the observed diversification in patterns of spatial self-organization.

To provide further evidence that neighborhood effects due to local differences in the initial spatial positionings of individuals can promote diversification in patterns of spatial self-organization, we performed mathematical simulations with an individual-based model that accounts for cell shoving during range expansion. The original model and its adaptions to range expansion are described in detail elsewhere [17, 53]. We further adapted the model to simulate the emergence of spatial self-organization during expansion of our own synthetic microbial community [20]. In this study, we applied the model for two purposes. First, we asked whether the model could simulate the simultaneous emergence of the two different patterns of spatial self-organization in the absence of spatial heterogeneity in the initial abiotic environment. Second, we varied the initial producer proportion and evaluated the consequences on the number of “consumer first” patterns that emerge during range expansion. Note that our implementation of the model does not incorporate genetic or stochastic phenotypic heterogeneity or demographic effects. However, our implementation does account for heterogeneity in the initial spatial positionings of individuals, as we randomly distributed individuals of the producer and consumer across the inoculation area prior to the onset of community expansion.

**Fig. 5:**
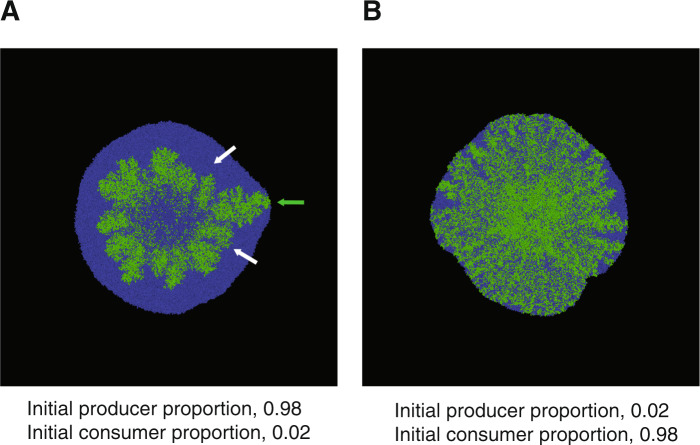
Individual-based modeling simulations of the effect of the initial producer proportion on the number of “consumer first” patterns that emerge per range expansion. The producer is blue while the consumer is green. Initial producer proportions are (**A**) 0.98 (see Supplementary Movie S1) and (**B**) 0.02 (see Supplementary Movie S2). The initial abiotic environment was spatially homogeneous and genetic heterogeneity, stochastic phenotypic heterogeneity, and demographic effects were not incorporated into the model. Producer and consumer cells were distributed randomly around the center prior to the onset of expansion and the total initial cell densities of producer and consumer were identical across all of the simulations. The white arrows indicate “producer first” patterns and the green arrow indicates a “consumer first” pattern.

Our simulations revealed three important outcomes. First, when we tested an initial producer proportion of 0.98 (i.e., an initial consumer proportion of 0.02), we found rare localized spatial areas where consumer cells were pushed forward by producer cells (Fig. 5A and Supplementary Movie S1). These cells maintained a spatial position at the expansion frontier for a prolonged period of time and formed a characteristic “consumer first” pattern (Fig. 5A and Supplementary Movie S1). Second, at this initial producer proportion, both “producer first” and “consumer first” patterns emerged simultaneously, from the very origin of expansion, and at the same length scale, even though the initial abiotic environment was spatially homogeneous and all individuals were subject the same deterministic rules (Fig. 5A and Supplementary Movie S1). Finally, when we decreased the initial producer proportion to 0.02 (i.e., an initial consumer proportion of 0.98), the number of consumer cells that maintained a position at the expansion frontier increased, thus indicating the formation of more “consumer first” patterns (Fig. 5B and Supplementary Movie S2). All three of these observations are consistent with our experimental observations and, importantly, did not require the consideration of heterogeneity in the initial abiotic environment, genetic or stochastic phenotypic heterogeneity within populations, or demographic effects. Thus, neighborhood effects due to local differences in the initial spatial positionings of individuals are sufficient alone to promote pattern diversification and result in the emergence of two different patterns of spatial self-organization.

### The different patterns of spatial self-organization have different community properties

We finally asked whether the different patterns of spatial self-organization have different community-level properties. More specifically, we tested whether the different patterns have different expansion speeds. Two features of our previous experimental observations already point towards this being the case. First, the “consumer first” patterns (green arrows; Fig. 1C) extend further in the radial direction of expansion than do the “producer first” patterns (white arrows; Fig. 1C). This is readily observed at the expansion frontier, where the “consumer first” patterns tend to protrude outwards in the radial direction (Fig. 1C). Second, the “consumer first” patterns increase in width in the direction of expansion (green arrows; Fig. 1C). Both of these features are consistent with faster expansion speeds [61, 62].

**Fig. 6:**
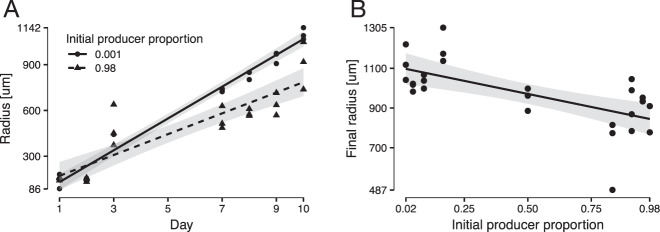
Effect of the initial producer proportion on expansion properties. **A** Effect on the initial expansion speed. **B** Effect on the final expansion radius. Each data point is the expansion radius for an independent range expansion. The lines are linear models and the gray areas are the 95% confidence intervals. The total initial cell densities of producer and consumer were identical across all of the tested initial producer proportions. The final expansion radii were measured after 4 weeks of incubation.

To further test this, we varied the initial producer proportion, and thus varied the ratio of “consumer first” to “producer first” patterns (Fig. 3), and quantified the expansion radii over time. We found that the initial expansion speeds were significantly faster for an initial producer proportion of 0.001 (i.e., an initial consumer proportion of 0.999) than for 0.98 (i.e., an initial consumer proportion of 0.02) (*F*-test; *P* = 1 × 10^−5^) (Fig. 6A). Thus, smaller initial producer proportions that promote the emergence of more ‘consumer first’ patterns result in faster expansion speeds. Moreover, when we varied the initial producer proportion between 0.02 and 0.98 (i.e., initial consumer proportions between 0.98 and 0.02), we found a decreasing relationship between the final expansion radius and the initial producer proportion (linear model: final expansion radius ~ initial producer proportion; slope = −263, *R*^2^ = 0.42, *P* = 2 × 10^−4^, *n* = 9) (Fig. 6B). Thus, smaller initial producer proportions that promote the emergence of more “consumer first” patterns result in a greater extent of community expansion over the time-course of the experiment. Together, our data demonstrate that the different patterns of spatial self-organization do indeed have different expansion speeds.

## Discussion

Using a combination of experiments, statistical modeling, and mathematical simulations, we demonstrated that multiple patterns of spatial self-organization can emerge at the same time and length scales in the absence of genetic heterogeneity within populations. Instead, we found that the simultaneous emergence of the different patterns can be explained by local differences in the initial spatial positionings of individuals that are otherwise subject to the same deterministic rules, and these local differences in initial spatial positionings can promote diversification in patterns of spatial self-organization. While we did not consider stochastic phenotypic heterogeneity or demographic effects as sources of nongenetic heterogeneity, the main point here is that local differences in the initial spatial positionings of otherwise identical individuals is sufficient alone to promote pattern diversification (Fig. 5). Thus, in order to predict patterns of spatial self-organization information, one may need information beyond the initial abiotic environment and the biological traits of individuals. Information about the initial spatial positionings of individuals and the physical/mechanical forces acting on those individuals may also be required.

Our results expand on a general concern regarding spatial pattern analysis in general. It is generally agreed upon that one cannot use spatial pattern analysis alone to conclusively infer interactions between different species or strains [63]. This is because different types of interactions and processes can, in principle, result in the emergence of qualitatively and even quantitatively similar patterns [63]. Our results here add even more caution to this concern. We show that even when a single interaction occurs between two species or strains in an abiotic environment that is initially spatially homogeneous, pattern diversification can occur and multiple patterns of spatial self-organization can emerge simultaneously (Figs. 2B, 5A). Thus, in order to conclusively infer interactions between species or strains, spatial pattern analysis likely must be combined with experimental manipulation and/or mathematical simulations that explicitly account for possible biological or abiotic heterogeneity, the physical/mechanical interactions acting on individuals, and the initial spatial positionings of those individuals.

Perhaps the most profound aspect of our study is that we identified a potentially general mechanism that creates a poorly understood type of biodiversity—spatial pattern diversity. Differences in the local spatial positionings of individuals can give rise to multiple patterns of spatial self-organization, and these different patterns can have different structural features and behavioral traits (Fig. 6). While biodiversity has long been known to be a critical factor determining community properties, such as community productivity [64–67], resilience and resistance to environmental change [68–71], and susceptibility to invasions [72–74], and has been formulated into the well-established theory of biodiversity–ecosystem functioning (BEF theory) [75–77], this body of research has largely focused on the taxonomic, phylogenetic, and/or functional aspects of biodiversity. We propose here that spatial pattern diversity is an important yet neglected aspect of biodiversity with regards to BEF theory, and its explicit consideration into BEF theory may generate novel insights and improve its general predictions.

How generalizable are our results? We believe that our main conclusions are potentially relevant to any surface-attached microbial community where individuals physically interact with each other during their growth and division. Such surface-attached microbial communities include many that are critically important to human health and our society, such as the communities that reside within in the human gut or are applied in industrial biofilm reactors. Importantly, at the initial inoculation stage of these surfaces, individuals will not be distributed evenly across space, and there will therefore be local differences in the initial spatial positionings of those individuals. Thus, the simultaneous emergence of multiple patterns of spatial self-organization and the creation of pattern diversity may be a pervasive, inevitable, and general feature of surface-attached microbial communities, with tangible consequences on predicting, managing, and controlling their properties and behaviors.

## Supplementary information

Supplementary Text

Supplementary Figure S1

Supplementary Figure S2

Supplementary Figure S3

Supplementary Figure S4

Supplementary Table S1

Supplementary Table S2

Supplementary Table S3

Supplementary Movie 1

Supplementary Movie 2
