# Supplementary material for: Causes and consequences of pattern diversification in a spatially self-organizing microbial community
Source: ISME J. 2021 Mar 4;15(8):2415–26. doi: 10.1038/s41396-021-00942-w (PMC8319339; doi:10.1038/s41396-021-00942-w)
Supplement: Supplementary file 1 — Supplementary Text [file 41396_2021_942_MOESM1_ESM.pdf]

Supplementary Text

Causes and consequences of pattern diversification in a spatially self-organizing microbial community

Felix Goldschmidt[1, 2], Lea Caduff[2], David R. Johnson[2,*]

[1]Department of Environmental Systems Science, Swiss Federal Institute of Technology (ETH), 8092 Zürich, Switzerland; [2]Department of Environmental Microbiology, Swiss Federal Institute of Aquatic Science and Technology (Eawag), 8600 Dübendorf, Switzerland.

*** -** Corresponding author (david.johnson@eawag.ch)

This file includes:

Extended Materials and Methods

Supporting Results

Supplementary Text References

Extended Materials and Methods

Strains used in this study. We provide a complete list of all the strains used in this study along with their genetic manipulations in Supplementary Table S1. We described all of the methods used to construct the strains in detail elsewhere [1-3].

Null expectations for the relationship between the initial producer proportion and the number of concurrent expansion patterns per range expansion. If we hypothesize that genetic variants of the producer cause the emergence of the concurrent expansion pattern, then we can derive a null expectation for the relationship between the initial producer proportion and the number of concurrent expansion patterns. We first assume that the initial frequency of the causative variant of the producer is constant within the total initial producer population. We therefore expect a proportional relationship between the initial abundance of the causative variant of the producer and the total initial abundance of the producer as presented in Equation 1, where $X_{v,P}$ is the initial abundance of the variant of the producer, $X_{T,P}$ is the total initial abundance of the producer, and c_1 is the frequency of $X_{v,P}$ within $X_{T,P}$.

Equation 1: $$X_{v,P} = c_1 \times X_{T,P}$$

We then assume that the number of concurrent expansion patterns that emerge is proportional to the initial abundance of the causative variant of the producer as presented in Equation 2, where y is the number of concurrent expansion patterns that emerge and C_2 is

the proportion of the causative variant of the producer that successfully causes a concurrent expansion pattern to emerge. Our justification for this assumption is that the concurrent expansion patterns emerge from the very origin of expansion (Fig. 1c), and the causative variant is therefore likely present in the initial population.

Equation 2: $y = c_2 \times X_{v,P} = c_2 \times c_1 \times X_{T,P}$

Finally, we rewrite Equation 2 in terms of the total initial abundance of the producer and consumer as presented in Equation 3, where X_T is the total initial abundance of the producer and consumer.

Equation 3: $\frac{y}{X_T} = c_2 \times c_1 \times \frac{X_{T,P}}{X_T}$

Thus, the null expectation is an increasing linear relationship between the number of concurrent expansion patterns that emerge per range expansion ($\frac{y}{X_T}$) and the initial producer proportion ($\frac{X_{T,P}}{X_T}$). The former quantity is the quantity that we experimentally measured while the latter quantity is the quantity that we experimentally manipulated.

Alternatively, if we hypothesize that genetic variants of the consumer cause the emergence of the concurrent expansion patter, then we can derive a similar null expectation for the relationship between the initial consumer proportion and the number of concurrent expansion patterns. We again expect a proportional relationship between the initial

abundance of the causative variant of the consumer and the total initial abundance of the consumer as presented in Equation 4, where $X_{v,C}$ is the initial abundance of the variant of the consumer, $X_{T,C}$ is the total initial abundance of the consumer, and c_3 is the frequency of $X_{v,C}$ within $X_{T,C}$.

Equation 4: $X_{v,C} = c_3 \times X_{T,C}$

We then again assume that the number of concurrent expansion patterns that emerge is proportional to the initial abundance of the causative variant of the consumer as presented in Equation 5, where y is the number of concurrent expansion patterns that emerge and C_4 is the proportion of the causative variant of the consumer that successfully causes a concurrent expansion pattern to emerge. Again, our justification for this assumption is that the concurrent expansion patterns emerge from the very origin of expansion (Fig. 1c), and the causative variant is therefore likely present in the initial population.

Equation 5: $y = c_3 \times X_{v,C} = c_4 \times c_3 \times X_{T,C}$

Finally, we rewrite Equation 5 in terms of the total initial abundance of the producer and consumer as presented in Equation 6, where X_T is the total initial abundance of the producer and consumer.

Equation 6: $\frac{y}{X_T} = c_4 \times c_3 \times \frac{X_{T,C}}{X_T}$

Thus, the null expectation is an increasing linear relationship between the number of concurrent expansion patterns that emerge per range expansion ($\frac{y}{X_T}$) and the initial consumer proportion ($\frac{X_{T,C}}{X_T}$). The former quantity is the quantity that we experimentally measured while the latter quantity is the quantity that we experimentally manipulated.

We can further rewrite Equation 6 in terms of the initial producer proportion rather than the initial consumer proportion by noting that the initial consumer proportion is equal to 1 – the initial producer proportion as depicted in Equation 7.

Equation 7: $\frac{y}{X_T} = c_4 \times c_3 \times \frac{1-X_{T,P}}{X_T} = -c_4 \times c_3 \times \frac{X_{T,P}}{X_T} + c_4 \times c_3$

Thus, the null expectation is a decreasing linear relationship between the number of concurrent expansion patterns that emerge per range expansion ($\frac{y}{X_T}$) and the initial producer proportion ($\frac{X_{T,P}}{X_T}$), which is the form presented in Fig. 3 in the main text.

Identifying putative genetic differences. To identify putative genetic differences that might have caused the emergence of the concurrent pattern of spatial self-organization, we performed non-target genomic DNA sequencing of four isolates of the producer and four isolates of the consumer isolated from independent prior concurrent expansion patterns. Briefly, we extracted genomic DNA using a Wizard Genomic DNA purification kit (Promega, Madison, WI) and digested contaminating RNA using RNAseA (5 Prime, Gaithersburg, MD). We then prepared sequencing libraries from the purified genomic DNA using the Nextera XT Sample Preparation and Nextera XT Index Kits (Illumina, San Diego, CA), added the PhiX Control v3 (Illumina, San Diego, CA) to the libraries, and sequenced the libraries using the MiSeq Reagent Kit v3 (Illumina, San Diego, CA) and a MiSeq sequencer (Illumina, San Diego, CA). We performed primary data analysis, de-multiplexing, and quality control analysis of the raw sequence reads using FastQC (Illumina, San Diego, CA). We quality filtered the raw sequence reads, removed duplicate sequence reads, and trimmed ambiguous base pairs using PRINSEQ-lite v0.20.4 [4]. We identified genetic differences between the genomes of each isolate and its corresponding reference strain using Breseq v.0.24rc5 and the utility program gdtools [5, 6]. Our analyses considered synonymous and non-synonymous point mutations, insertions, deletions, and multiplications. We reported all of the parameters for identifying genetic differences elsewhere [7]. We summarized all of the putative genetic differences in Supplementary Table S2. We deposited all of the raw sequences to the NCBI Sequence Read Archive (https://www.ncbi.nlm.nih.gov/sra) under BioSample accessions SAMN12631003 to SAMN12631010.

We used Sanger sequencing [8] to independently validate the putative genetic differences identified from the MiSeq (Illumina, San Diego, CA) genomic DNA sequencing effort. We designed primers to target specific putative genetic differences identified by MiSeq sequencing (Illumina, San Diego, CA) (Supplementary Table S3), amplified the target regions using PCR amplification, and verified that the PCR products had the expected length using conventional agarose gel electrophoresis. We then gel-purified the PCR products using the Wizard SV Gel and PCR Clean-Up System (Promega, Madison, WI), quantified the concentrations of the purified PCR products using a Nanodrop spectrophotometer (Thermo Fisher Scientific, Waltham, MA) or a Qbit fluorometer (Thermo Fisher Scientific, Waltham, MA), and sent the PCR products to Microsynth (Balgach, Switzerland) for Sanger sequencing.

Individual-based model simulations. We simulated the growth of the producer using Equation 8 and the consumer using Equation 9 as reported previously [9]. The equations use a Monod-type term for growth with the appropriate substrate along with an inhibition term that increases with increasing nitrite (NO_2^-) concentration as described elsewhere [1]. For this study, however, we grew the communities under conditions where nitrite does not have any observable inhibitory effects [1]. We therefore set the $K_{inhibition}$ term to a sufficiently high level such that there was negligible nitrite inhibition. The parameter values are provided in Supplementary Table S2.

Equation 8: $$\mu_{Producer} = \mu_{max,Producer} \cdot \frac{S_{NO3}}{K_{NO3} + S_{NO3}} \cdot \frac{K_{inhibition}}{K_{inhibition} + S_{NO2}}$$

Equation 9: $$\mu_{Consumer} = \mu_{max,Consumer} \cdot \frac{S_{NO2}}{(1 + i \cdot S_{NO3}) \cdot K_{NO2} + S_{NO2}} \cdot \frac{K_{inhibition}}{K_{inhibition} + S_{NO2}}$$

In these simulations, we set μ_{max} to 1 for both the producer and consumer as reported previously [9]. While there are slight differences in the growth rates of the producer and consumer [2], these slight differences are not the main factor driving spatial self-organization in our experimental system, and they do not qualitatively affect our modelling simulations. This is because the main factor driving spatial self-organization is the temporal segregation in the initiation of growth with nitrate (NO_3^-) and nitrite (NO_2^-) [2, 9]. The producer can grow immediately regardless of its relative growth rate to the consumer for two reasons. First, only nitrate is initially present. Second, nitrate reduction begins immediately without any observable lag [2]. In contrast, the consumer only grows after the producer releases a sufficient amount of nitrite to support growth. Moreover, even when nitrite is present, the consumer has a short lag (1-2h) before reaching its maximum growth rate [2]. These effects are accounted for with the term $(1 + i \cdot S_{NO3})$ in Equation 9. We described and discussed this term in detail in previous manuscripts [1, 9].

Supporting Results

The 'producer first' and 'consumer first' patterns emerge as a consequence of the genetically engineered cross-feeding interaction. We have three lines of evidence that the 'producer first' and 'consumer first' patterns emerge as a consequence of nitrite (NO_2^-) cross-feeding between the producer and consumer.

1. We previously analyzed and compared the patterns formed by two complete denitrifiers and by the producer and consumer [2]. The two complete denitrifiers

form a fundamentally pattern, which consists of sectors that have interspecific boundaries lying approximately parallel to the axis of expansion and extending from the inoculation zone to the edge of the expansion zone. In contrast, the producer and consumer form the canonical ‘producer first’ and ‘consumer first’ patterns as reported here.

2. We performed an experiment where we grew the producer and consumer with an exogenous supply of both nitrate (NO_3^-) and nitrite (NO_2^-) (Supplementary Fig. S1). This enables both strains to grow independently, thus breaking the metabolic interaction between them. We found that the ‘producer first’ and ‘consumer first’ patterns disappeared (Supplementary Fig. S1). Instead, the producer and consumer segregate into sectors as we reported previously when we imposed competition between two complete denitrifiers [2]. However, while the producer and consumer largely segregate into sectors, there is more lateral intermixing than we observed for competition between two complete denitrifiers (*i.e.*, the inter-specific boundaries are not as straight). This suggests that, while nitrite is provided exogenously, there may nevertheless be some nitrite cross-feeding occurring behind the expansion frontier.

3. We performed an experiment where we grew the producer and consumer under aerobic conditions (Supplementary Fig. S2). Under these conditions, the producer and consumer compete for oxygen, thus breaking the metabolic interaction between them. We found that the ‘producer first’ and ‘consumer first’ patterns again disappeared (Supplementary Fig. S2). Instead, the producer and consumer segregate

into sectors similar to those that we reported previously when wee imposed competition between two complete denitrifiers [2].

Taken together, these three lines of evidence provide a solid basis for concluding that the 'producer first' and 'consumer first' patterns do indeed emerge as a consequence of the genetically engineered cross-feeding interaction between the producer and consumer.

Analysis of putative genetic differences. To assess whether the concurrent pattern of spatial self-organization has a genetic basis, we sequenced the genomes of four producer isolates and four consumer isolates that we purified from independent prior concurrent expansion patterns. We then determined whether those isolates contain genetic differences when compared to their respective ancestors. To accomplish this task, we used a combination of MiSeq and Sanger sequencing. We first used MiSeq sequencing to detect putative genetic changes and then used Sanger sequencing to verify those putative genetic changes. When using MiSeq sequencing, we detected a number of putative genetic differences (Supplementary Table S3). However, when using Sanger sequencing, we could only validate one of the tested putative genetic differences in one of the consumer isolates (Supplementary Table S3). We then used Sanger sequencing to check again whether this specific mutation was present in any of the other strains, but did not find it in any of them. We therefore conclude that this specific mutation could not be the cause of the concurrent expansion pattern. All of the other tested putative genetic differences were either already present in the respective ancestors or were sequencing errors (*i.e.*, MiSeq sequencing detected a putative genetic difference while Sanger sequencing did not) (Supplementary

Table S3). Our results therefore suggest that the concurrent expansion pattern is not caused by genetic variants within the producer or consumer.

Supplementary Text References

1. Lilja EE, Johnson DR. Segregating metabolic processes into different microbial cells accelerates the consumption of inhibitory substrates. ISME J. 2016;10:1568-78.
2. Goldschmidt F, Regoes RR, Johnson DR. Successive range expansion promotes diversity and accelerates evolution in spatially structured microbial populations. ISME J. 2017;11:2112-23.
3. Lilja EE, Johnson DR. Metabolite toxicity determines the pace of molecular evolution within microbial populations. BMC Evol Biol. 2017;17:52
4. Schmieder R, Edwards R. Quality control and preprocessing of metagenomic datasets. Bioinformatics. 2011;27:863-4.
5. Barrick JE, Lenski RE. Genome-wide mutational diversity in an evolving population of *Escherichia coli*. Cold Spring Harb Symp Quant Biol. 2009;74:119-29.
6. Barrick JE et al. Genome evolution and adaptation in a long-term experiment with *Escherichia coli*. Nature. 2009;461:1243-7.
7. Marchal M et al. A passive mutualistic interaction promotes the evolution of spatial structure within microbial populations. BMC Evol Biol. 2017;17:106.
8. Sanger F, Coulson AR. A rapid method for determining sequences in DNA by primed synthesis with DNA polymerase. J Mol Biol. 1975;94:441-8.
