# Supplementary material for: Causes and consequences of pattern diversification in a spatially self-organizing microbial community
Source: ISME J. 2021 Mar 4;15(8):2415–26. doi: 10.1038/s41396-021-00942-w (PMC8319339; doi:10.1038/s41396-021-00942-w)
Supplement: Supplementary file 2 — Supplementary Figure S1 [file 41396_2021_942_MOESM2_ESM.pdf]

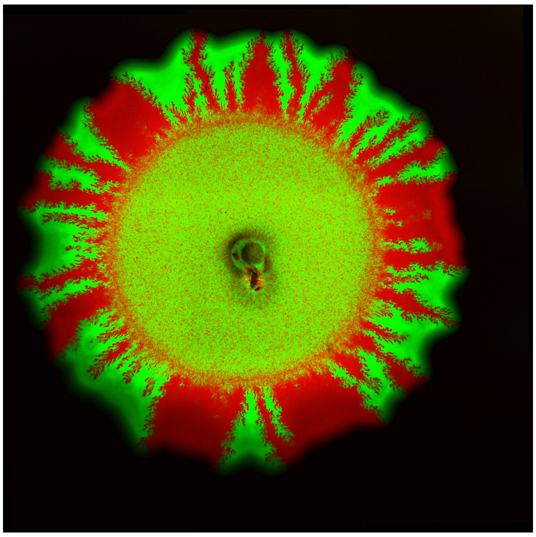

Supplementary Fig. S1: Expansion of the producer and consumer when provided with an exogenous supply of both nitrate and nitrite. The producer expressed the red fluorescent protein-encoding *echerry* gene (red) while the consumer expressed the green fluorescent protein-encoding *egfp* gene (green). The initial producer and consumer proportions were 0.5. Note that an exogenous supply of nitrate and nitrite eliminates the metabolic dependence between the two strains and that the 'producer first' and 'consumer first' patterns no longer emerge.
