# Supplementary material for: Causes and consequences of pattern diversification in a spatially self-organizing microbial community
Source: ISME J. 2021 Mar 4;15(8):2415–26. doi: 10.1038/s41396-021-00942-w (PMC8319339; doi:10.1038/s41396-021-00942-w)
Supplement: Supplementary file 3 — Supplementary Figure S2 [file 41396_2021_942_MOESM3_ESM.pdf]

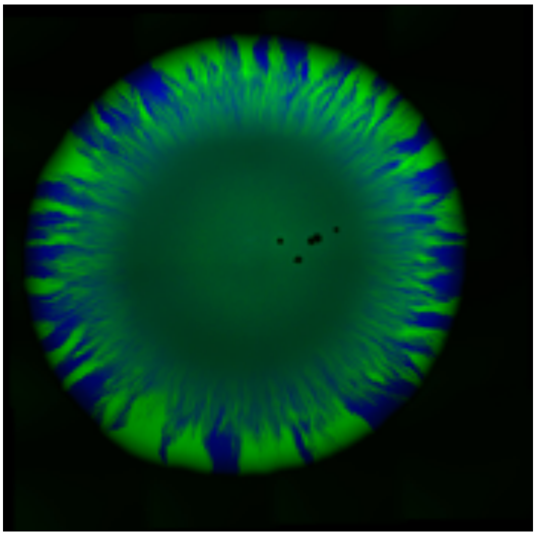

Supplementary Fig. S2: Expansion of the producer and consumer when provided with oxygen and not with nitrate. The producer expressed the cyan fluorescent protein-encoding *ecfp* gene (blue) while the consumer expressed the green fluorescent protein-encoding *egfp* gene (green). The initial producer and consumer proportions were 0.5. Note that providing oxygen rather than nitrate eliminates the metabolic dependence between the two strains and that the 'producer first' and 'consumer first' patterns no longer emerge.
