# Supplementary material for: Causes and consequences of pattern diversification in a spatially self-organizing microbial community
Source: ISME J. 2021 Mar 4;15(8):2415–26. doi: 10.1038/s41396-021-00942-w (PMC8319339; doi:10.1038/s41396-021-00942-w)
Supplement: Supplementary file 4 — Supplementary Figure S3 [file 41396_2021_942_MOESM4_ESM.pdf]

A **B**

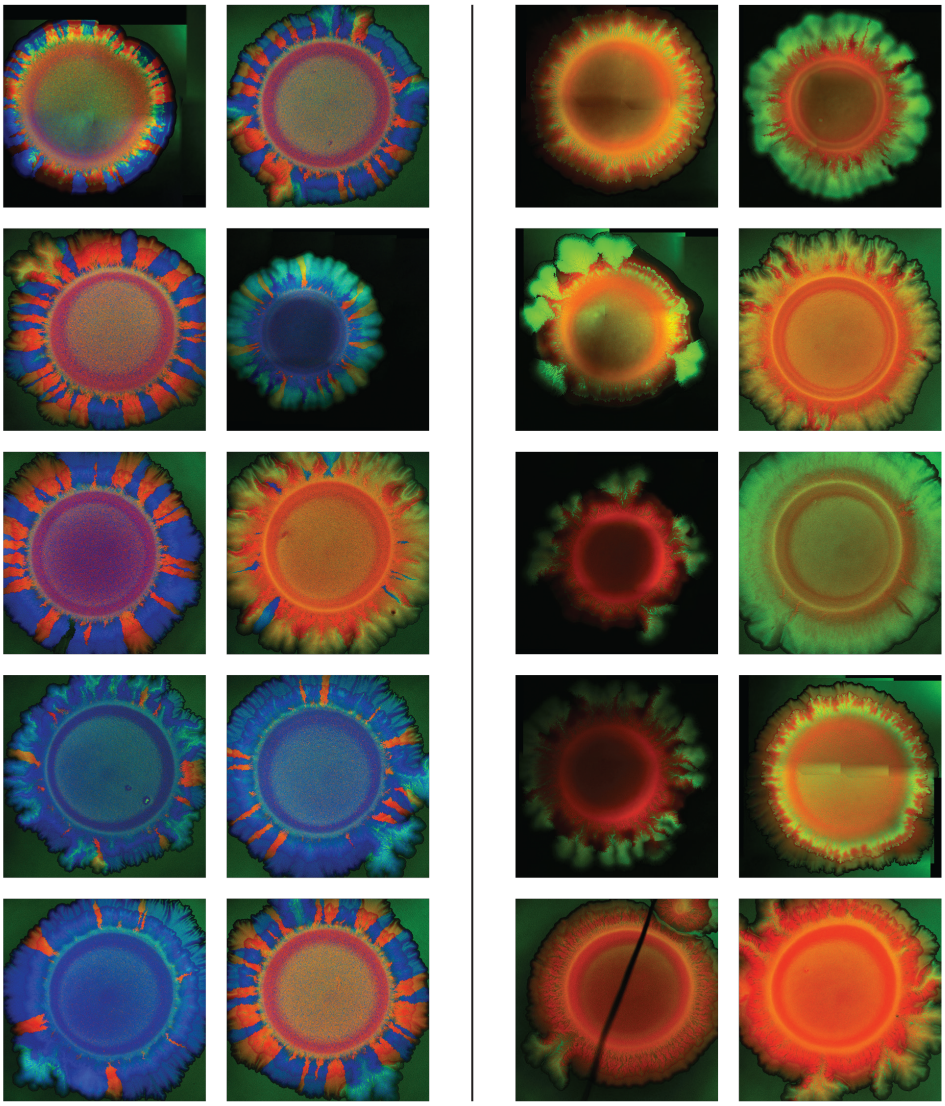

Supplementary Fig. S3: Effect of the origin of the producer and consumer on the patterns of spatial self-organization that emerge during range expansion. The composition of the community is **a** ancestral pairs of producer (blue and red) and consumer (green), or **b** pairs of producer (red) and consumer (green) isolated from prior 'consumer first' patterns. Note that we used two strains of the producer when performing these experiments with the ancestral strain. This has no impact on the results, as the two producer strains (blue and red) have identical growth properties (note the straight boundaries between the two strains).
