# Supplementary material for: Causes and consequences of pattern diversification in a spatially self-organizing microbial community
Source: ISME J. 2021 Mar 4;15(8):2415–26. doi: 10.1038/s41396-021-00942-w (PMC8319339; doi:10.1038/s41396-021-00942-w)
Supplement: Supplementary file 5 — Supplementary Figure S4 [file 41396_2021_942_MOESM5_ESM.pdf]

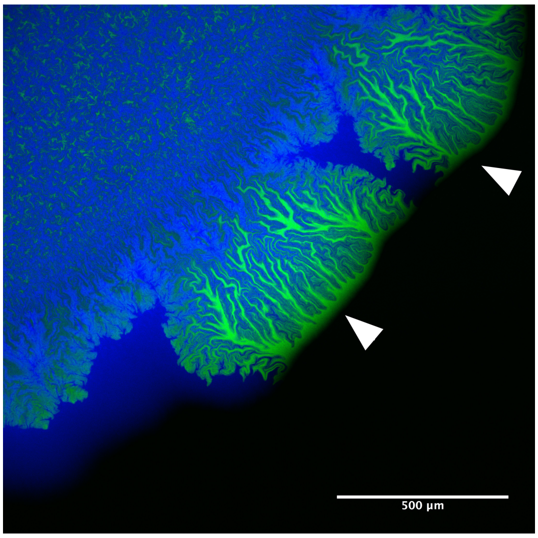

Supplementary Fig. S4: Twelve days of expansion of the producer and consumer when supplied with nitrate as the growth-limiting substrate. The producer expressed the cyan fluorescent protein-encoding *ecfp* gene (blue) while the consumer expressed the green fluorescent protein-encoding *egfp* gene (green). The initial producer and consumer proportions were 0.5. White arrows indicate representative regions where the consumer is pushed forward by the producer.
