# Supplementary material for: Causes and consequences of pattern diversification in a spatially self-organizing microbial community
Source: ISME J. 2021 Mar 4;15(8):2415–26. doi: 10.1038/s41396-021-00942-w (PMC8319339; doi:10.1038/s41396-021-00942-w)
Supplement: Supplementary file 6 — Supplementary Table S1 [file 41396_2021_942_MOESM6_ESM.pdf]

Supplementary Table S1: Strains used in this study.

Pseudomonas stutzeri strain	Relevant characteristics	Supplementary Text Reference
A1601gfp	A1501 with Δ*comA* and mini-Tn7T-LAC-Gm-*egfp*; Gm^R, $egfp^+$	1-3
A1601ech	A1501 with Δ*comA* and mini-Tn7T-LAC-Gm-*echerry*; Gm^R, $echerry^+$	1-3
A1601cfp	A1501 with Δ*comA* and mini-Tn7T-LAC-Gm-*ecfp*; Gm^R, $ecfp^+$	1-3
A1602gfp	A1502 with Δ*comA*, Δ*narG* and mini-Tn7T-LAC-Gm-*egfp*; Gm^R, $egfp^+$	1-3
A1602ech	A1502 with Δ*comA*, Δ*narG* and mini-Tn7T-LAC-Gm-*echerry*; Gm^R, $echerry^+$	1-3
A1602cfp	A1502 with Δ*comA*, Δ*narG* and mini-Tn7T-LAC-Gm-*ecfp*; Gm^R, $ecfp^+$	1-3
A1603gfp	A1503 with Δ*comA*, Δ*nirS* and mini-Tn7T-LAC-Gm-*egfp*; Gm^R, $egfp^+$	1-3
A1603ech	A1503 with Δ*comA*, Δ*nirS* and mini-Tn7T-LAC-Gm-*echerry*; Gm^R, $echerry^+$	1-3
A1603cfp	A1503 with Δ*comA*, Δ*nirS* and mini-Tn7T-LAC-Gm-*ecfp*; Gm^R, $ecfp^+$	1-3
