# Supplementary material for: Causes and consequences of pattern diversification in a spatially self-organizing microbial community
Source: ISME J. 2021 Mar 4;15(8):2415–26. doi: 10.1038/s41396-021-00942-w (PMC8319339; doi:10.1038/s41396-021-00942-w)
Supplement: Supplementary file 7 — Supplementary Table S2 [file 41396_2021_942_MOESM7_ESM.pdf]

Supplementary Table S2: Parameter values of the individual based model.

Parameter	Value
μ_{max}	1
Yield	0.5 g biomass/g substrate
K_{NO3}, K_{NO2}	0.35 mg/L
Competitive substrate inhibition *i*	1.5 L/mg
$K_{inhibition}$	$1x10^{20}$ mg/L
Diffusion constant NO_3^-, NO_2^-	200 mm^2/h
Initial NO_3^- concentration	250 mg/L
System size	600 µm
Total initial number of cells	4000
