# Supplementary material for: Causes and consequences of pattern diversification in a spatially self-organizing microbial community
Source: ISME J. 2021 Mar 4;15(8):2415–26. doi: 10.1038/s41396-021-00942-w (PMC8319339; doi:10.1038/s41396-021-00942-w)
Supplement: Supplementary file 8 — Supplementary Table S3 [file 41396_2021_942_MOESM8_ESM.pdf]

Supplementary Table S3: Putative genetic differences identified by Illumina sequencing for isolates purified from prior concurrent expansion patterns.

Clone	Genome position	Genetic change	Annotation[a]	Gene	Confirmation with Sanger sequencing
Producer 1	3,515,718	G->C	non-coding	23S rRNA	tested, false detection
	3,515,720	G->A	non-coding	23S rRNA	tested, false detection
	3,515,729	C->T	non-coding	23S rRNA	tested, false detection
	3,515,731	C->G	non-coding	23S rRNA	tested, false detection
	3,515,773	A->G	non-coding	23S rRNA	tested, false detection
Producer 2	382,561	T->C	non-synonymous (C138R)	*pip*	tested, false detection
	1,584,385	Δ2647 bp	deletion	PST_1476-1479	tested, false detection
	2,161,809	Δ60 bp	deletion	PST_1992	tested, false detection
	3,515,718	G->C	non-coding	23S rRNA	tested, false detection
	3,515,720	G->A	non-coding	23S rRNA	tested, false detection
	3,515,729	C->T	non-coding	23S rRNA	tested, false detection
	3,515,731	C->G	non-coding	23S rRNA	tested, false detection
	3,515,773	A->G	non-coding	23S rRNA	tested, false detection
Producer 3	382,561	T->C	non-synonymous (C138R)	*pip*	tested, false detection
	1,584,385	Δ2647 bp	deletion	PST_1476-1479	tested, false detection
	3,515,718	G->C	non-coding	23S rRNA	tested, false detection
	3,515,720	G->A	non-coding	23S rRNA	tested, false detection
	3,515,729	C->T	non-coding	23S rRNA	tested, false detection
	3,515,731	C->G	non-coding	23S rRNA	tested, false detection
	3,515,773	A->G	non-coding	23S rRNA	tested, false detection
Producer 4	382,561	T->C	non-synonymous (C138R)	*pip*	tested, false detection
	1,393,981	A->G	intergenic	PST_1284->/->PST_1285	tested, false detection
	1,584,385	Δ2647 bp	deletion	PST_1476-1479	tested, false detection
	2,161,809	Δ60 bp	deletion	PST_1992	tested, false detection
	2,392,899	+T	coding	*mtlK*	tested, false detection
	3,515,720	G->A	non-coding	23S rRNA	tested, false detection
	3,515,729	C->T	non-coding	23S rRNA	tested, false detection
	3,515,731	C->G	non-coding	23S rRNA	tested, false detection
	3,515,773	A->G	non-coding	23S rRNA	tested, false detection
	3,875,178	G->A	synonymous (R78R)	PST_3583	tested, false detection
Consumer 1	2,161,809	Δ60 bp	deletion	PST_1992	tested, false detection
	2,392,899	+T	coding	*mtlK*	tested, false detection
	3,515,773	A->G	non-coding	23S rRNA	tested, false detection
	3,668,036	Δ36,873 bp	RP3-mediated	PST_3393-3428	tested, false detection
Consumer 2	1,501,527	C->T	non-synonymous (P1340L)	PST_1402	tested, confirmed
	2,161,809	Δ60 bp	deletion	PST_1992	tested, false detection
	3,515,729	C->T	non-coding	23S rRNA	tested, false detection
	3,515,731	C->G	non-coding	23S rRNA	tested, false detection
	3,515,773	A->G	non-coding	23S rRNA	tested, false detection
Consumer 3	3,515,729	C->T	non-coding	23S rRNA	tested, false detection
	3,515,731	C->G	non-coding	23S rRNA	tested, false detection

	3,515,773	A->G	non-coding	23S rRNA	tested, false detection
Consumer 4	2,392,899	+T	coding	*mtlK*	tested, false detection
	3,515,718	G->C	non-coding	23S rRNA	tested, false detection
	3,515,720	G->A	non-coding	23S rRNA	tested, false detection
	3,515,729	C->T	non-coding	23S rRNA	tested, false detection
	3,515,731	C->G	non-coding	23S rRNA	tested, false detection
	3,515,773	A->G	non-coding	23S rRNA	tested, false detection

[a]The type of mutation and, if applicable, type of amino acid change detected in each clone. Alphabetic letters indicate amino acid residues.
